# A Multiple Sensors Platform Method for Power Line Inspection Based on a Large Unmanned Helicopter

**DOI:** 10.3390/s17061222

**Published:** 2017-05-26

**Authors:** Xiaowei Xie, Zhengjun Liu, Caijun Xu, Yongzhen Zhang

**Affiliations:** 1 Institute of Photogrammetry and Remote Sensing, Chinese Academy of Surveying and Mapping, Beijing 100830, China; 2014102140006@whu.edu.cn (X.X.); yz_zhang87@163.com (Y.Z.); 2 School of Geodesy and Geomatics, Wuhan University, Wuhan 430079, China; cjxu@sgg.whu.edu.cn

**Keywords:** unmanned helicopter, power line inspection, automatic target tracking

## Abstract

Many theoretical and experimental studies have been carried out in order to improve the efficiency and reduce labor for power line inspection, but problems related to stability, efficiency, and comprehensiveness still exist. This paper presents a multiple sensors platform method for overhead power line inspection based on the use of a large unmanned helicopter. Compared with the existing methods, multiple sensors can realize synchronized inspection on all power line components and surrounding objects within one sortie. Flight safety of unmanned helicopter, scheduling of sensors and exact tracking on power line components are very important aspects when using the proposed multiple sensors platform, therefore this paper introduces in detail the planning method for the flight path of the unmanned helicopter and tasks of the sensors before inspecting power lines, and the method used for tracking power lines and insulators automatically during the inspection process. To validate the method, experiments on a transmission line at Qingyuan in Guangdong Province were carried out, the results show that the proposed method is effective for power line inspection.

## 1. Introduction

With the development of the global economy and population, the power demand keeps rising, leading to an increasing scale of expansion of overhead transmission lines, of which a major part needs to cross regions with relatively adverse natural environment and complex terrain, such as rivers, mountains, forests and even depopulated zones, etc. Because the power lines and towers suffer from long-term exposure to the environmental conditions in the field, combining the influences of material aging, thunder strikes, and interference from trees, it is highly possible for malfunctions of electric power equipment to happen, including broken conductors, damaged or overheating insulators, tilted towers, and discharges caused by trees [1,2]. Therefore electric power departments need to conduct regular inspection on electric transmission lines, to detect potential abnormal conditions and to conduct timely maintenance.

The current inspection on electric transmission lines mainly depends on manpower inspection and manned helicopter inspection. However the labor intensity is quite high, the working conditions are poor and the inspection efficiency concerning manpower inspection is low; whereas in the case of manned helicopter inspections, the cost is quite high, and the operation is risky. In recent years, a variety of schemes have been proposed to replace the manual inspection and manned helicopter inspection, which can be divided into three categories. The first category is the use of various kinds of robots for power line inspection [3,4,5,6,7,8,9], for example, the “LineScout” developed by the Hydro-Québec research institution (Varennes, QC, Canada), the “Expliner” developed by the Tokyo Institute of Technology (Tokyo, Japan), the “TI” developed by Electric Power Research Institute (Palo Alto, CA, USA), etc. These robots are characterized by high safety and low cost, can be used in continuous inspection for fault diagnosis of power lines, the deficiency is that they cannot support inspection on other power line components and surrounding objects. The second category is inspection methods using cameras or video cassette recorders (VCRs) based on unmanned aerial vehicles (UAVs); examples can be seen in [10,11,12,13,14,15,16,17,18,19]. Power line components and surrounding objects can be inspected by using different sensors in different sorties. However, limited by the short flight time of UAVs, it is unfeasible to conduct long-distance inspection on power lines. The third category is inspection methods using typical photogrammetric or LiDAR techniques based on manned aerial vehicles, which are more effective and can be used for long distance inspection, but the details of the power line components cannot be obtained by these methods [20,21,22,23].

In this paper, we propose a multiple sensors platform method for power line inspection. Compared to the schemes mentioned above, the use of multiple sensors can support inspection of all power line components and surrounding objects within one sortie. A large unmanned helicopter is used here to meet the requirements for carrying the multiple sensors platform and to support the power line inspection over a long distance. Therefore, it will be more effective and more comprehensive for power line inspection.

When the multiple sensors platform is used for power line inspection, the flight safety of the unmanned helicopter, scheduling of sensors and exact tracking of power line components are very important aspects. This paper present the method of using a multiple sensor platform, which includes two steps, the first is to plan the flight path of the unmanned helicopter and the coordinates needed to perform the tasks for each sensor; the second is to complete all tasks automatically based on an exact target tracking algorithm, through which we can acquire different data about power line components or surrounding objects efficiently and accurately with different sensors during the inspection process.

This paper is organized as follows: Section 2 introduces the components of the multiple sensors platform. Section 3 introduces the planning for the flight paths of the unmanned helicopter and the tasks of each sensor before inspecting the power lines. Section 4 introduces the automatic tracking method used during the power line inspection process. Section 5 validates the dependability of the method presented in this paper on the basis of experiments, followed by conclusions in Section 6.

## 2. The Composition of the Multiple Sensors Platform

**Figure 1:**
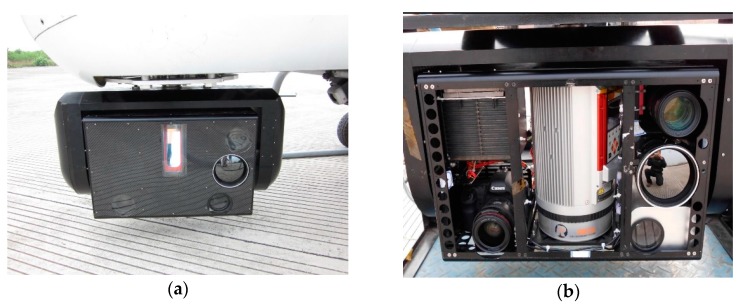
Appearance of the multiple sensors platform: (**a**) Overall picture of the multiple sensors platform; (**b**) Appearance of the sensor pod.

The multiple sensors platform includes two parts, inertial stabilized platform and the sensor pod, as shown in Figure 1a. A two-axis inertial stabilized platform is adopted here [24], which is used to adjust the attitude of the sensor pod so that the sensors’ line of sight (LOS) can track the targets accurately in real time. The sensor pod is the unit where all the sensors are placed, which integrates six sensors in total including the position and orientation system (POS), light detection and ranging (LiDAR), thermal camera, ultraviolet camera, a long-focus camera and a short-focus camera as shown in Figure 1b. Among all the sensors, the lines-of-sight of the infrared camera and the ultraviolet camera, the long-focus camera and the short-focus camera are parallel to each other.

POS can obtain the real-time attitude and coordinates of the sensor pod, and it is used on real-time target tracking during the power line inspection and on the data analysis after the inspection. The high-precision point cloud acquired by LiDAR can be used on monitoring the distance between the electric transmission lines and the surroundings (trees in particular). The thermal infrared data collected by the thermal camera can be used on monitoring the heat status of facilities including electric power fittings, power lines and insulators, etc. The ultraviolet videos acquired by the ultraviolet camera can be used for monitoring abnormal discharges of electric power fittings, power lines and insulators. Images acquired by the short-focus camera can be used on inspecting whether there are broken conductors in the shield wires and titled towers. High-resolution images acquired by the long-focus camera can be used on diagnosing the anomalies including missing pins, burst insulators and corroded electric power fittings. Parameters of the sensors are shown in Table 1, including sensor name, product model, focal length, field of view (FOV), and data type.

## 3. Planning of Flight Paths and Tasks of the Sensors

The planning of the flight paths of the unmanned helicopter and tasks of the sensors includes two parts: the first is to design the flight paths of the unmanned helicopter while inspecting the electric transmission lines; and the second is to plan the tasks of all sensors while the unmanned helicopter is flying along the flight paths. The current UAV power lines inspection generally adopts one sensor, the flight path planning mostly involves connecting and smoothing a series of flight-path segments referring to the towers of power lines, which fails to consider other influencing factors, and the sensor mostly continue working on time-lapse, so there is no need to plan sensor tasks [25,26,27]. Different from the current flight paths planning, the flight control system of the unmanned helicopter presented in this paper can automatically start the smoothing function according to the waypoints, so we only need to provide waypoints with no smoothing. In addition, while planning the waypoints, we also take into account the influence of complex terrain (such as mountains) and non-target power lines. On the other hand, using multiple sensors can support inspection for all power line components and surrounding objects within one sortie, so scheduling of sensors is very important aspect. Therefore, this paper presents the planning method for the tasks of all sensors during the inspection process.

Before planning the flight paths and tasks of the sensors, it is assumed that the high-precision 3D point cloud of the entire electric transmission lines has been acquired (the LiDAR on the multiple sensors platform can be used to acquire the point clouds), thus the precise 3D coordinates of the towers, insulators and power lines can be obtained, as well as the digital surface model (DSM) data of the entire transmission line corridor, of which the resolution and elevation accuracy are 15 m and 3 m, respectively.

### 3.1. Waypoints Planning of the Unmanned Helicopter

There are three reasons for planning the waypoints of the unmanned helicopter. First, the unmanned helicopters have a maximum flight time which is limited by their fuel carrying capacity. Second, the distance between the unmanned helicopter and the power lines must be larger than a safe distance. Finally, the closer the unmanned helicopter is to the electric transmission lines, the better the data collected by the sensors is, and to better acquire data from insulators on each tower, it is necessary to slow down at each tower. Therefore, it is necessary to plan the waypoints in a way that can not only guarantee a safe and effective inspection, but also ensure data quality good. The planning of the waypoints of using the unmanned helicopter on inspecting electric transmission lines includes three steps. The first step is to create the referential waypoints according to coordinates of the towers of the electric transmission lines that need to be inspected. The second step is to optimize the referential waypoints based on the DSM data and coordinates of non-target electric transmission lines. Finally, based on the waypoints of unmanned helicopter, operating parameters of LiDAR can be calculated.

#### 3.1.1. Referential Waypoints Planning

**Figure 2:**
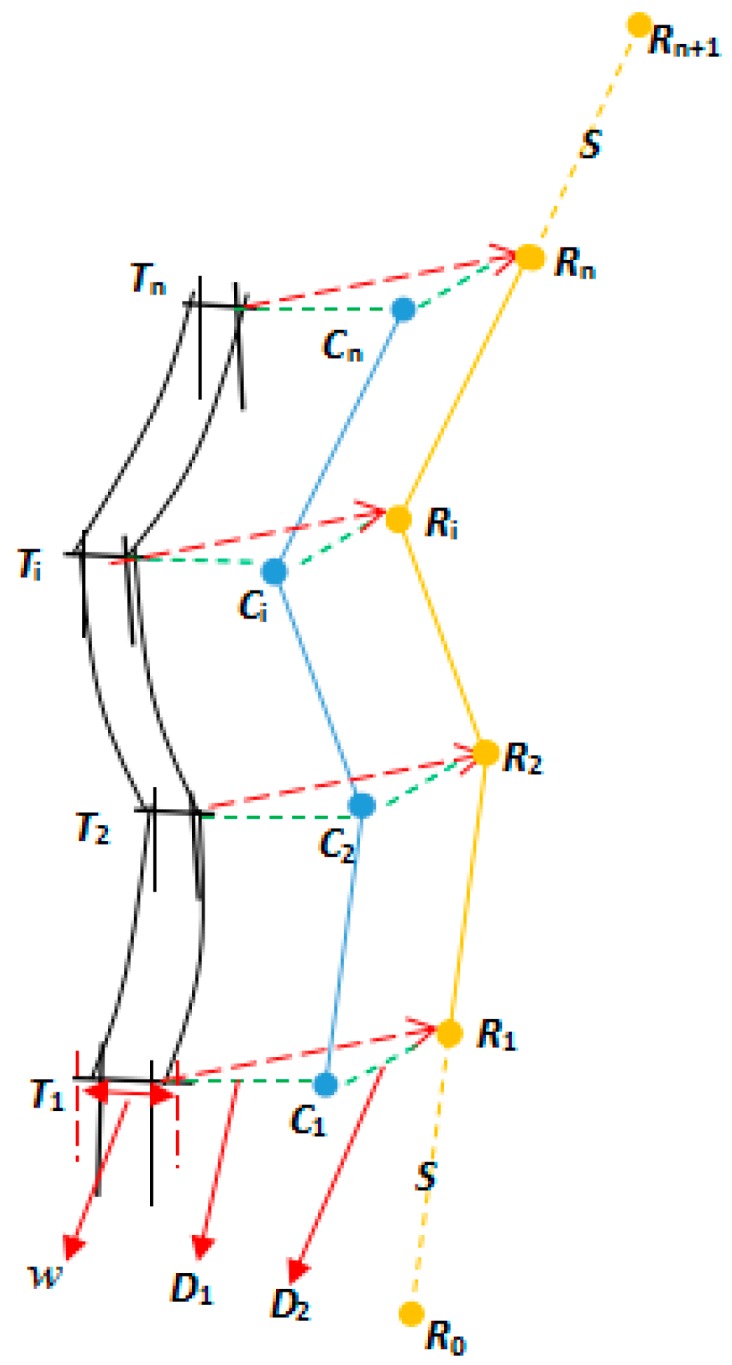
The principle of referential waypoints planning.

Assuming that $D_{1}$ is the horizontal safe distance between the unmanned helicopter and the power lines, $D_{2}$ is the vertical safe distance between the unmanned helicopter and the power lines. As shown in Figure 2, T(x,y,z,w) representing the n towers on the electric transmission lines, (x,y,z,w) representing for the centric coordinates at the top and the width of the towers, these data can be acquired from the 3D point cloud data of the electric transmission lines. We begin by connecting the points of every two towers to obtain a series of line segments $\bar{T_{1}T_{2}},\bar{T_{2}T_{3}},\cdots ,\bar{T_{n-1}T_{n}}$. Secondly, move all the line segments with $D_{1}+w/2$ horizontally to obtain a series of cross points $C=\left\{C_{1},C_{2},\cdots ,C_{n}\right\}$, then move C with $D_{2}$ vertically to obtain the temporary waypoints, which is $R^{\prime }=\left\{R_{1},R_{2},\cdots ,R_{n}\right\}$. Finally, extend the distance S along $\bar{R_{2}R_{1}}$ to obtain point $R_{0}$ as the inlet point, and extend the distance S along $\bar{R_{n-1}R_{n}}$ to obtain point $R_{n+1}\;$as the appearance point, the $R=\left\{R_{0},R_{1},\cdots ,R_{n},R_{n+1}\right\}$ are the final waypoints, S is set according to the practical situation.

#### 3.1.2. Optimization of Waypoints

Unavoidably, except for target electric transmission lines that need to be inspected, sometimes there are also non-target transmission lines in the same area. Therefore, we also need to ensure the safety distance between the unmanned helicopter and the non-target transmission lines. In addition, given the complex environment of the transmission line corridor, it is also need to keep a safe distance between the unmanned helicopter and ground to guarantee the safety of the unmanned helicopter. We assume that $D_{3}$ is the safe distance between the unmanned helicopter and the ground.

**Figure 3:**
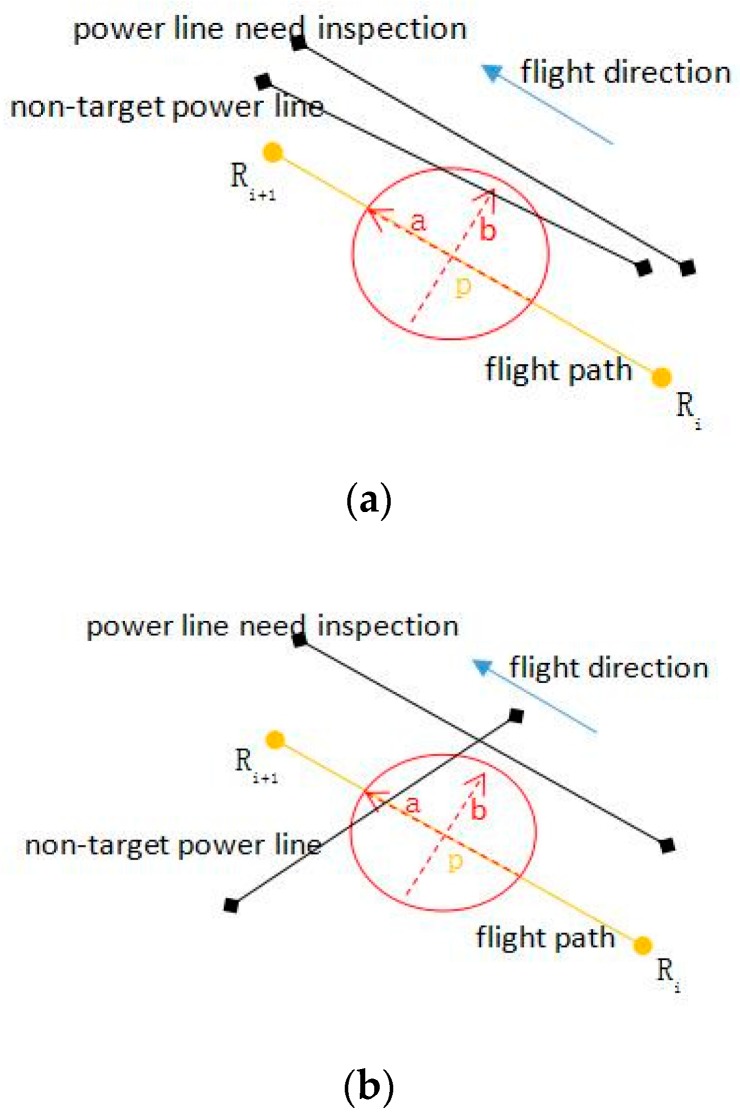
The interference of non-target power lines: (**a**) Power lines in parallel; (**b**) Power lines cross over.

The optimization of waypoints is to check and handle security risks in referential waypoints, based on the DSM data and coordinates of non-target transmission lines. The detailed steps of optimizing the waypoints are as follows. For the auxiliary waypoints obtained in the following steps, the unmanned helicopter does not slow down at these waypoints, therefore here each flight-path segment $\bar{R_{i}R_{i+1}}$ is deems as a whole, which the auxiliary waypoints are included in, as $\bar{R_{i}\cdots R_{p}\cdots R_{i+1}}$:

(a) For any one segment$\;\bar{R_{i}R_{i+1}}$, disperse it into n temporary points according to a step length.
(b) Staring from$\;R_{i}$, we take each temporary point p as the center and $D_{3}$ as the radius to draw a circle. Based on the DSM data, estimate whether the distance between the coordinates of point p and the ground within the circle is greater than$\;D_{3}$ is estimated. If it is greater, the following steps should be continued; if it is less, the auxiliary waypoint$\;R_{p}$ is added at point p. The height of$\;R_{p}\;$should enable the distance between$\;R_{p}\;$and all ground points within the circle to be greater than$\;D_{3}$. When point p is too close to a certain waypoint, this waypoint can be heightened to ensure that the height of point p meets the requirement, without the necessity to adding new auxiliary waypoints.
(c) Taking point p as the center and taking$\;D_{1}\;$as the radius, re-draw a circle to estimate whether there are non-target power lines falling into the new circle, as shown in Figure 3.
(d) If there are non-target power lines fall into the new circle and cross with axle b, the distance between point p and the power lines is $r^{\prime }$, it need to add an auxiliary waypoint$\;R_{p}$ acquired by moving p outwardly with $D_{1}-r^{\prime }$.
(e) If there are power lines fall into this circle and cross with axle a, it is necessary to estimate whether the vertical distance between point p and the power line is greater than$\;D_{2}$. If it is greater, nothing needs to be done; if it is less, it is necessary to add the auxiliary waypoint$\;R_{p}$, of which the (x,y) can be acquired by intersection point between the power lines and axle a, and z is the height of the power lines added with$\;D_{2}$. Similarly, when point p is too close to a certain waypoint, it is feasible to directly heighten this waypoint to ensure that the safe distance between point p and the lines can meet the requirement.
(f) Repeat Steps b–e to complete the optimization of all waypoints. It needs to be noted that, once a new auxiliary waypoint was added in steps b, d, and e, it is necessary to recalculate the dispersed temporary points from the last waypoint, then repeat Steps b–e.

#### 3.1.3. Operating Parameters of LiDAR

In order to ensure the data acquired by LiDAR meet the requirement, reasonable operating parameters need to be calculated before power line inspection. The longitudinal and lateral distances between laser footprints ($d_{lon},d_{lat}$) can be calculated according to the following formulas:

(1) $$d_{lon}=v/2f_{s}$$

(2) $$d_{lat}=4hf_{s}\tan\left(\theta /2\right)/F$$

(3) $$h=\sqrt{D_{1}^{2}+D_{2}^{2}}$$

(4) $$\theta =\tan^{-1}\left(\left(B-D_{1}\right)/H\right)+\tan^{-1}\left(\left(B+D_{1}+w^{\prime }\right)/H\right)$$

(5) $$\theta_{s}=\pi /2+\tan^{-1}\left(\left(D_{1}+w^{\prime }/2\right)/D_{2}\right)-\tan^{-1}\left(\left(B+D_{1}+w^{\prime }\right)/H\right)$$

(6) $$\theta_{e}=\theta_{s}+\theta$$

**Figure 4:**
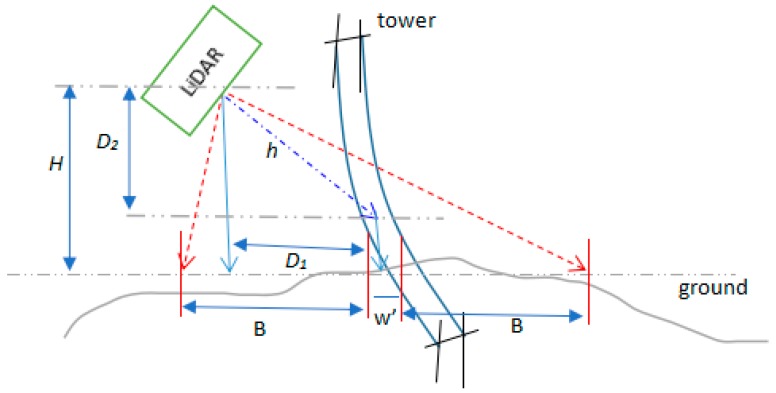
The scanning angle of LiDAR.

In the formulas above, v represents flight speed of the unmanned helicopter, $f_{s}$ represents scanning speed, h represents distance between LiDAR and power lines, F represents pulse repetition rate, θ represents scanning angle, B represents the distance need to be covered on each side of the power lines, $w^{\prime }$ represents the average width of the power lines, H represents the average flying height relative to the ground, $\theta_{s}$ represents starting scanning angle, and $\theta_{e}$ represents terminational scanning angle, as shown in Figure 4. According to the requirement of $d_{lon}$ and $d_{lat}$ for power line inspection, $\left(f_{s},F,\theta_{s},\theta_{e}\right)$ can be determined.

### 3.2. Planning of Tasks of the Sensors

Task planning is the realization of the scheduling of the sensors. The LiDAR, thermal camera and ultraviolet camera can start work directly when the unmanned helicopter reaches the starting point and can stop while it reaches the ending point; it only needs to set task points at the starting point and ending point for starting and stopping data collection. Therefore the major part of the planning of tasks of the sensors is to plan the task points for the short-focus camera to track the power lines and the long-focus camera to track the insulators. Task points represent the initial position for the cameras to carry out the photo-taking tasks; each task point corresponds to a target position. Here the infrared camera and the ultraviolet camera are not taken into consideration, because the lines-of-sight of the thermal camera, ultraviolet camera, short-focus cameras and long-focus cameras are parallel to each other.

**Figure 5:**
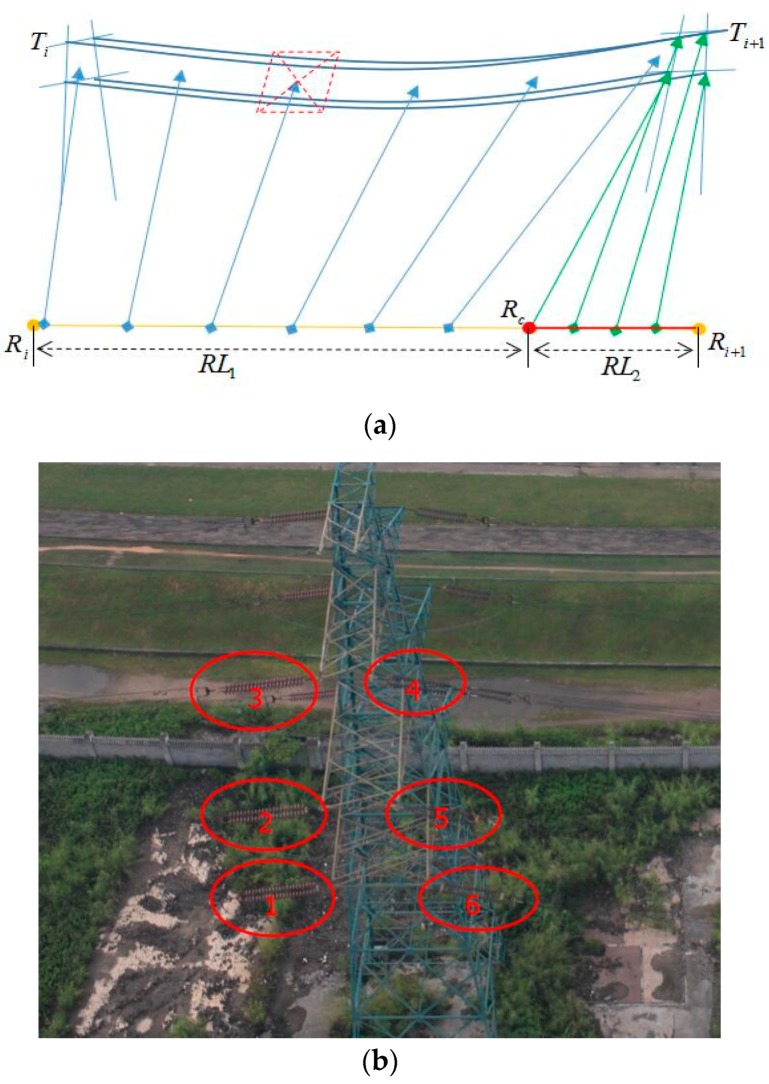
The principle of task planning: (**a**) The layout of task points for two cameras; (**b**) The order to shoot insulators of the long-focus camera.

As shown in Figure 5a, assuming that $\bar{T_{i}T_{i+1}}$ is a segment of power lines composed of two neighboring towers, and the length is TL. The corresponding flight segment of the unmanned helicopter is $\bar{R_{i}R_{i+1}}$, and the length is RL, $R_{c}$ is a temporary point close to $R_{i+1}$.

To ensure the short-focus camera and the long-focus camera can accomplish all tasks when the unmanned helicopter is flying through the flight segment, task points for the short-focus camera to track the power lines of $\bar{T_{i}T_{i+1}}$ are set at $\bar{R_{i}R_{c}}$ and task points for the long-focus camera to track the insulators on the $T_{i+1}$ towers are set at $\bar{R_{c}R_{i+1}}$. The detailed steps are as follows:

(a) According to the insulators’ number of $T_{i+1}$ towers on the right side, we can get the number M of the task points for the long-focus camera. The centric coordinates of the insulators are used as target positions, and the shooting order is from bottom to top and from left to right as shown in Figure 5b. The insulators on the other side of the towers are inspected through bilateral flying.
(b) To ensure that there is enough time for the implementation, set the minimum distance between every two task points as ts, multiplied by M, we can get $RL_{2}=ts\times M\;$which is the flight distance of the unmanned helicopter when the long-focus camera is tracking the insulators. The start point$\;R_{c}\;$of the task points of the long-focus camera can be obtained based on$\;RL_{2}$, and the spacing between the two task points is ts.
(c) Calculate the image width W according to FOV of the short-focus camera and the distance between camera and power lines, the distance here adopts $D_{1}+w/2$, which is mentioned in Section 3.1. Starting from$\;T_{i}$, calculate the times the short-focus camera need to take photos between $\bar{T_{i}T_{i+1}}$ in the horizontal direction as the overlap is q, N=TL/((1−q)×W)+1. The centric position of each image can be used as the target position for tracking.
(d) Then to plan uniformly-spaced task points for the short-focus camera according to N on the flight segment of $\bar{R_{i}R_{c}}$, the length of $\bar{R_{i}R_{c}}$ is $RL_{1}$. However, when RL<(N+M)×ts, all the task points of the short-focused camera need to be completed starting from$\;R_{i}$ based on a separation distance of ts. The task points of the long-focus camera are put off orderly, and the exceeded task points are all set in the position of $R_{i+1}$. Meanwhile the unmanned helicopter is set to hovering at the position of $R_{i+1}$, and the hovering duration is determined according to the amount of the exceeded tasks and the minimum duration needed to implement the tasks.

According to the steps above, we can obtain the coordinates of all task points in the flight segment of $\bar{R_{i}R_{i+1}}$ and the corresponding targets.

## 4. Automatic Target Tracking

This paper adopts the double closed loop control method to achieve the automatic target tracking. The outer loop adopts the distance control method, enabling the camera to start aiming and tracking when the helicopter is close enough to the task point, to ensure the orderly implementation of the inspection tasks. The inner loop adopts the attitude control method, which uses the real-time coordinates of the projection center of the camera obtained from POS and the coordinates of the targets to be tracked to calculate the pitch and heading, adjust the posture of camera by gradually adjusting the stabilized platform, which meets the requirements when the difference between posture of camera and the pitch and heading we calculated is small enough. Real-time DGPS is adopted here to ensure the precision of the coordinates of the camera.

**Figure 6:**
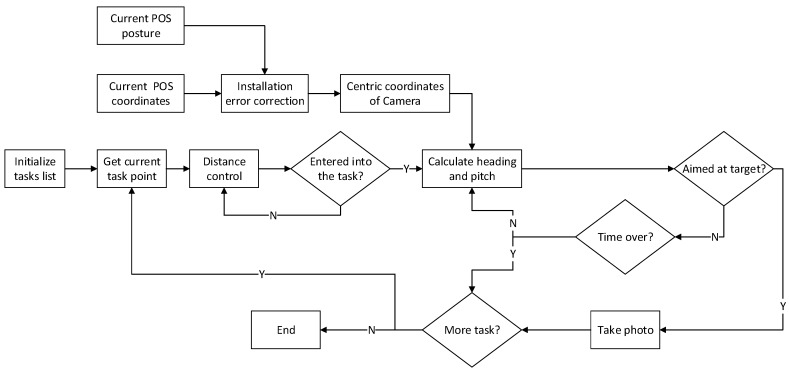
The principle of automatic target tracking.

The principle of algorithm is as shown in Figure 6. The first step is to obtain the coordinates of the current task points through initializing the task list. Second is to check whether enter into the current task in real time according to the distance control method. Third is to calculate the heading and pitch angle of the target through the coordinates of the target points and the centric coordinates of the camera which are corrected by installation errors, and send the angles to the platform to adjust the posture of camera. Last is to estimate in real time whether the current attitude of the camera meets the requirements for photo-taking, and send the instruction for take photo to the camera and continue the next task if it meets the requirements.

One important thing to note about this method is that there is a time limit for the execution of each task because the distance between each two task points is fixed based on the tasks planning. Therefore, once the task has not been completed within the specified time, it will be given up and the next task will be started. There are three critical parts in the entire task implementation, which are real-time correction of installation errors, distance control and attitude control, the details in Section 4.1, Section 4.2 and Section 4.3.

### 4.1. Real-time Correction of Installation Errors

During the inspection process on power lines, real-time coordinates of the phase center of the GPS antenna output by POS are obtained directly. To ensure the precision of the automatic tracking, it is necessary to transfer the real-time coordinates of the phase center of the GPS antenna to the real-time coordinates of the photographing center of the cameras by correcting the installation errors:

(7) $$\left[\begin{array}{c} x_{c}^{e}\\ y_{c}^{e}\\ z_{c}^{e} \end{array}\right]=R_{n}^{e}\left(L,B\right)R_{l}^{n}\left(R,P,H\right)R_{g}^{l}\left(\alpha ,\beta ,\gamma \right)\left[\left[\begin{array}{c} \Delta x_{g}^{w}\\ \Delta y_{g}^{w}\\ \Delta z_{g}^{w} \end{array}\right]+R_{c}^{g}\left(\varphi ,\omega ,k\right)\left[\begin{array}{c} \Delta x_{w}^{c}\\ \Delta y_{w}^{c}\\ \Delta z_{w}^{c} \end{array}\right]\right]+\left[\begin{array}{c} x_{e}\\ y_{e}\\ z_{e} \end{array}\right]$$

In the equation above, $R_{n}^{e}\left(L,B\right)\;$is the rotation matrix from the navigation coordinate system to the geocentric coordinate system. $R_{l}^{n}\left(R,P,H\right)$ is the rotation matrix from the IMU coordinate system to the navigation coordinate system. $R_{g}^{l}\left(\alpha ,\beta ,\gamma \right)$ is the rotation matrix from the reference coordinate system of the stabilized platform to the IMU coordinate system. $R_{c}^{g}\left(\varphi ,\omega ,k\right)$ is the rotation matrix from the camera coordinate system to the reference coordinate system of the stabilized platform. $\left(x_{c}^{e},y_{c}^{e},z_{c}^{e}\right)$ is the 3D coordinates of the photographing center. $\left(x_{e},y_{e},z_{e}\right)$ is the GPS coordinates. $\left(\Delta x_{g}^{w},\Delta y_{g}^{w},\Delta z_{g}^{w}\right)$ is the eccentric component between the center of the stabilized platform and the phase center of the GPS antenna. $\left(\Delta x_{w}^{c},\Delta y_{w}^{c},\Delta z_{w}^{c}\right)$ is the eccentric component from the photographing center of the camera to the center of the stabilized platform.

### 4.2. Distance Control

**Figure 7:**
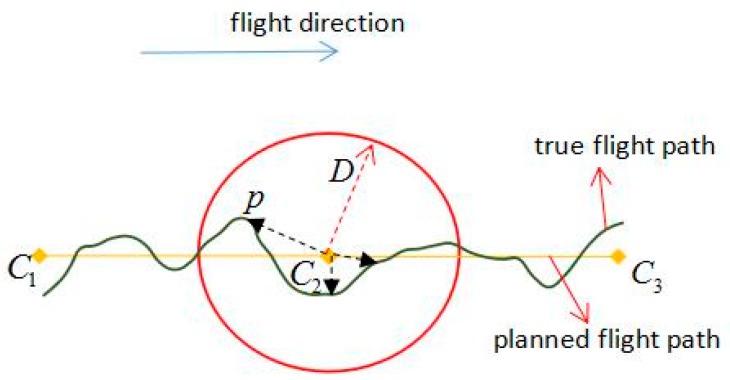
The principle of distance control.

As shown in Figure 7, the unmanned helicopter has to pass the task points planned in Section 3.2 in ideal conditions. However, influenced by various factors, the unmanned helicopter cannot fly strictly according to the planned flight paths, causing difficulties in estimating directly whether the position of the unmanned helicopter satisfies the conditions to implement the tasks. The usual solution is to set a distance threshold D. When the distance between the unmanned helicopter and the task point is less than D, it meets the requirement, but the value of D here is not easy to set, when the value of D is too small and the unmanned helicopter is too deviated from the planned flight paths, the possibility of failing to implement the tasks is quite high; and when the value of D is too great, the tracking effect will be influenced. This paper present a method combining distance and angle to check whether it is satisfied with the requirement to perform tasks or not.

It is assumed that the current position of the unmanned helicopter is p, the location of the last task point is $C_{1}$, and the location of the next task will be carried out is $C_{2}$. Firstly a greater distance threshold D is set and we ensure that the distance between p and $C_{2}$ will certainly meet the threshold D. Secondly, calculate $∠pC_{2}C_{1}$ when the distance between p and $C_{2}$ is smaller than D, From Figure 6 it can be seen that when $∠pC_{2}C_{1}$ reaches 90°, the unmanned flies over the cross section of $C_{2}$ exactly. Lastly, an angle threshold is set $\theta <3^{{}^{\circ}}$, it is satisfied with the requirement when p meets $\left|90^{{}^{\circ}}-∠pC_{2}C_{1}\right|\leq \theta$, $∠pC_{2}C_{1}$ cannot reach $90^{{}^{\circ}}$ exactly due to data errors.

### 4.3. Attitude Control

After the position of the unmanned helicopter meets the requirements for implementing the current task, we calculate the heading and the pitch angle between the photographing center of the camera and the target, and send adjustment instruction to the stabilized platform. When the difference between the posture of camera and the calculated angle smaller than angle threshold, we perform the current task. The steps of the attitude control are as follows:

(a) Motion compensation: If the time-delay of the camera shooting is t, before calculating the heading and the pitch angle between the photographing center of the camera and the target, it needs to take the distance of the unmanned helicopter flying forward within t into consideration. The current coordinates of the photographing center of the cameras are $\left(x_{0},y_{0},z_{0}\right)$, the current speeds provided by POS are $\left(v_{x},v_{y},v_{z}\right)$, and after the time t, the photographing center of the cameras reach the coordinates of $\left(x_{1},y_{1},z_{1}\right)$. According to Equation (8), $\left(x_{1},y_{1},z_{1}\right)$ can be obtained:
  (8) $$\left[\begin{array}{c} x_{1}\\ y_{1}\\ z_{1} \end{array}\right]=R_{l}^{c}\left(\alpha^{\prime },\beta^{\prime },\gamma^{\prime }\right)\left[\begin{array}{c} v_{x}t\\ v_{y}t\\ v_{z}t \end{array}\right]+\left[\begin{array}{c} \Delta x\\ \Delta y\\ \Delta z \end{array}\right]+\left[\begin{array}{c} x_{0}\\ y_{0}\\ z_{0} \end{array}\right]$$
  In the formula above, $R_{l}^{c}\left(\alpha^{\prime },\beta^{\prime },\gamma^{\prime }\right)$ is the rotation matrix from the IMU coordinate system to the coordinate system of the camera. (Δx,Δy,Δz) is the eccentric component between the IMU center and the center of the camera.
(b) Angle calculation: Coordinates of the photographing center of the camera are $\left(x_{1},y_{1},z_{1}\right)$; coordinates of the target are $\left(x_{2},y_{2},z_{2}\right)$. We can calculate the heading and pitch at according to Equations (9) and (10):
  (9) $$\mathrm{heading}=\{\begin{array}{c} \pi /2-\varepsilon \;\left(x_{2}>x_{1},y_{2}\neq y_{1}\right)\\ 3\pi /2-\varepsilon \left(x_{2}<x_{1},y_{2}\neq y_{1}\right)\\ 0\;\left(x_{2}=x_{1},y_{2}>y_{1}\right)\\ \pi \;\left(x_{2}=x_{1},y_{2}<y_{1}\right) \end{array}$$
  (10) $$\mathrm{pitch}=\sin^{-1}\left(\left(z_{1}-z_{2}\right)/dist\right)$$
  (11) $$k=\left(y_{2}-y_{1}\right)/\left(x_{2}-x_{1}\right)$$
  (12) $$\varepsilon =\tan^{-1}\left(k\right)$$
  (13) $$dist=\sqrt{{\left(x_{2}-x_{1}\right)}^{2}+{\left(y_{2}-y_{1}\right)}^{2}+{\left(z_{1}-z_{2}\right)}^{2}}$$
(c) Attitude control: To send angle adjustment instructions to the stabilized platform. Setting an angle threshold to conduct real-time estimation on whether the attitude of the platform meets the requirement for the threshold value. If it meets the requirement, photographing instructions are sent to the cameras; if it does not meet the requirement, we repeat Steps a-c until the requirement is met.

## 5. Experiments and Analyses

**Figure 8:**
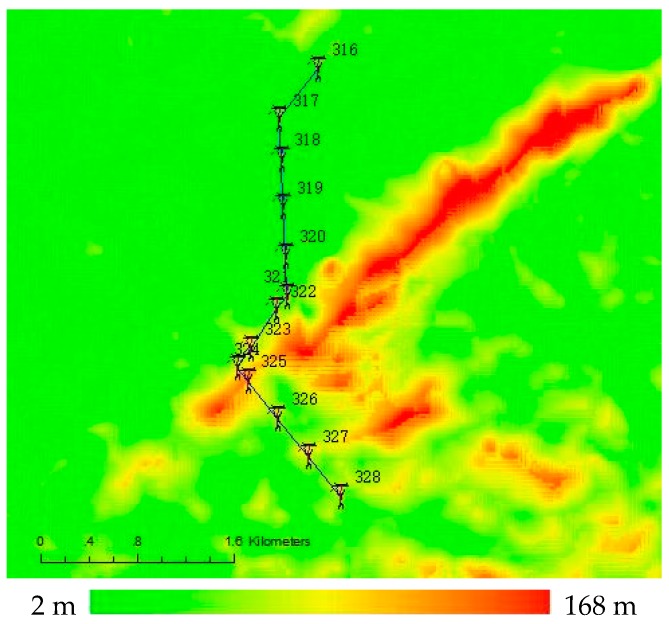
Appearance of power line for the experiments. The elevation in this area is color-coded.

A 110 kV electric transmission line at Qingyuan in Guangdong Province was selected for experiments to validate the feasibility of the method presented in this paper. The total length of this line is 4.2 km; it has 13 towers that are numbered from 316 to 328, and has 78 insulators. The geographical environment of the power line passages is complex with quite great topographic relieves, as shown in Figure 8. The 3D point cloud data of this electric transmission line was obtained by APLIS. We adopted the method introduced in Part 3 to plan the waypoints of unmanned helicopter and the tasks of sensors. The operating Parameters of LiDAR are shown in Table 2, and the parameters adopted in the planning are as shown in Table 3.

**Figure 9:**
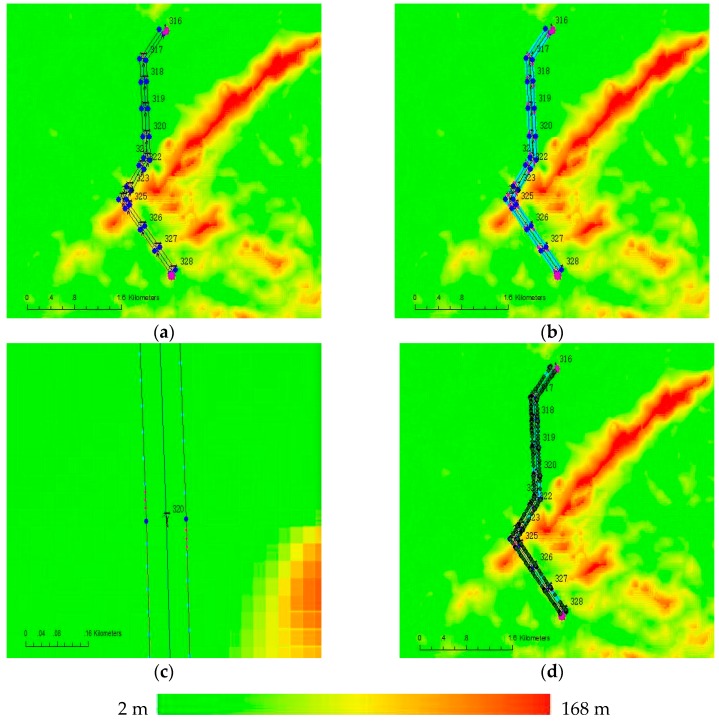
The result of planned flight path and tasks, and true flight path, the elevation in this area is color-coded. (**a**) The planned flight path; (**b**) The planned task points; (**c**) The zoomed image of task points at tower No. 320; (**d**) The true flight path during power line inspection.

Figure 9a shows the planned waypoints of the unmanned helicopter. There are 30 waypoints, of which the green ones are starting points and the purple ones are ending points. After taking off from around tower No. 328, the unmanned helicopter firstly flew along the right side of the power lines from tower No. 328 to tower No. 316, then it flew along the left side of the power lines from tower No. 316 to tower No. 328. At a place near tower No. 323, there is a crossing between a non-target power line and the experimental power line, so we increase the height of the corresponding waypoint. In addition, we add an auxiliary waypoint between the two waypoints corresponding the tower No. 324 and tower No. 325, for the large terrain variation. Figure 9b presents the task points of the cameras. For the short-focus camera, there are 163 task points which are displayed in light blue, and the average distance between each two task points is about 30 m. For the long-focus camera, there are 78 task points which are displayed in pink, and the distance between each two task points is 5 m. The distance between every two towers is long enough, therefore the unmanned helicopter does not need to hover at any one of towers to extend the time to take photos of the insulators. Figure 9c shows the task points at tower No. 320. Figure 9d presents the comparison between the actual flight paths and the planned flight paths of the unmanned helicopter, the deep green represents for the actual waypoints, and it can be seen that the unmanned helicopter cannot strictly fly according to the planned flight paths. The line-pressing accuracy in this experiment is about ±5 m.

Data acquired in this experiment includes 956 M laser-point cloud data of the entire passages of the power lines, 15.8 G HD infrared videos of the electric transmission line, 51.4 G ultraviolet videos of the electric transmission line, 163 images of the power lines taken by the short-focus camera, and 73 images of the insulators taken by the long-focus camera.

The scheduling method (task planning of sensors) of multiple sensors can be evaluated by the success rates of the tasks. Compared with the planned task points, it can be found that the success rate of tasks for the short-focus camera and the long-focus camera are 100% and 93.5% respectively, as shown in Table 4. From the Section 4, we know that the distance between each two task points will limit the time for task execution, therefore there are several failed tasks for the long-focus camera affected by some factors (adjusting speed of the stable platform, response time of camera, and flight speed of unmanned helicopter, etc.), while the success rate of tasks for the short-focus camera is higher than the long-focus camera because the distance between each two task points of which is longer. However, longer distance between each two task points for the long-focus camera will affect the image resolution and viewing angle, so we should take a balance between data quality and task execution time based on actual power lines information when conducting power line inspection.

**Figure 10:**
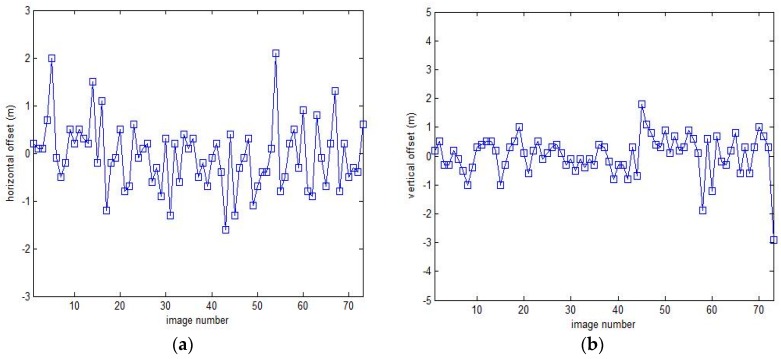
Offset between image center and the target. (**a**) Offset in horizontal direction; (**b**) Offset in vertical direction.

The effect of automatic target tracking can be evaluated through analyzing and comparing the offset distance between the photo center and the target object on the images. Because the FOV of the long-focus camera is smaller than the short-focus camera and it is more difficult to tacking target for the long-focus camera, we use the result of the long-focus camera to evaluate the effect of automatic target tracking. Figure 10 shows the horizontal and vertical offsets between the photo center and the target object on the images respectively, the mean values are −0.05 m and 0.04 m, and the standard deviation are 0.71 m and 0.69 m, as shown in Table 5. However, the image coverage is 6.7 m in the horizontal direction and 10 m in the vertical direction, greater than the offset distance, so we can find targets in all images. Thus we can conclude that the automatic target tracking method perform quite well.

**Figure 11:**
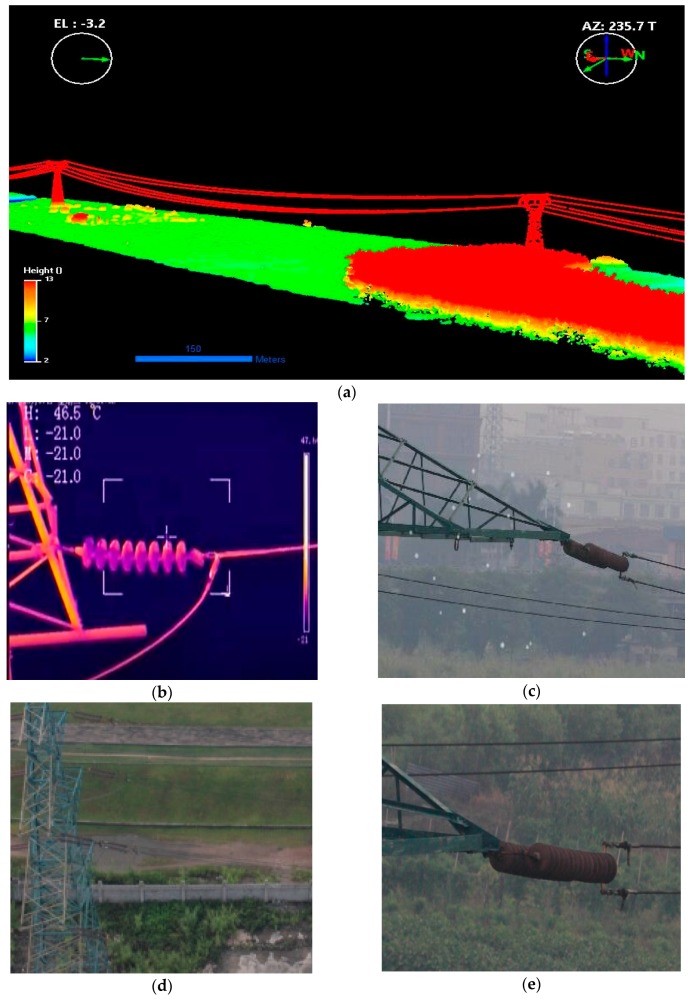
The data acquired by sensors during power line inspection. (**a**) The LiDAR point cloud, the compass on the left displays elevation, while the compass on the right displays azimuth; (**b**) The thermal infrared data, H, L, M and C represent the maximum temperature, minimum temperature, temperature at image center, and temperature at crosshair position respectively; (**c**) The photons acquired by ultraviolet camera, superimposed on the optical image; (**d**) The image of tower acquired by the short-focus camera; (**e**) The zoomed image of the insulator acquired by the long-focus camera.

Figure 11 shows the data acquired by multiple sensors platform. Figure 11a shows the point cloud data of power lines acquired by LiDAR, the compass on the left displays elevation, while the compass on the right displays azimuth; Figure 11b shows the thermal infrared data acquired by thermal camera, H, L, M and C represent the maximum temperature, minimum temperature, temperature at image center, and temperature at crosshair position respectively; Figure 11c shows the photons acquired by ultraviolet camera, which are superimposed on the optical image; Figure 11d shows the image of tower acquired by short-focus camera; Figure 11d shows a zoomed image of an insulator acquired by the long-focus camera.

**Figure 12:**
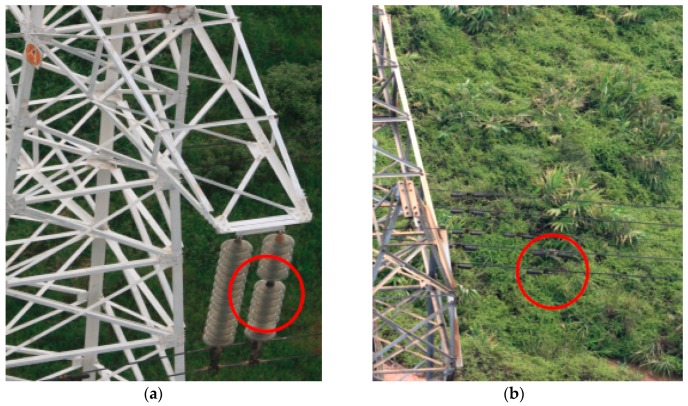
Two anomalies on the power line. (**a**) Broken insulator on tower No. 319; (**b**) A torsional dampers fallen off at tower No. 327.

Different data acquired by sensors can be synchronized based on POS data. After manual analysis of the data, two anomalies were found, the first is a broken insulator on tower No. 319, another is that one of the torsional dampers had fallen off at tower No. 327, as shown in Figure 12.

## 6. Conclusions

In this paper, a multiple sensors platform method based on the use of a large unmanned helicopter was proposed for power line inspection. LiDAR, thermal camera, ultraviolet camera, and two cameras with different focal lengths are used to acquire information about power line components and surrounding objects. Scheduling of sensors, exact tracking on power line components and flight safety when the multiple sensors platform is used to power line inspection were introduced in detail. Experiments were carried out on an 110 kV electric transmission line at Qingyuan in Guangdong Province, and the results show that the proposed method is effective for power line inspection. In the future, the method needs to be optimized to further improve the success rate and accuracy of the task execution. In addition, we will also start research on the automatic processing and analysis of data acquired by multiple sensors platform for power line inspection.

## Figures and Tables

**Table 1:** Parameters of the sensors.

Sensor Name	Product Model	Focal Length (mm)	FOV (H×V)	Data Type
LiDAR	Rigel VZ400	-	-	Point cloud
Thermal camera	customized	100	$9^{{}^{\circ}}\times 6^{{}^{\circ}}$	Video
Ultraviolet camera	customized	-	$9^{{}^{\circ}}\times 6.75^{{}^{\circ}}$	Video
The short-focus camera	Canon 5D Mark II	35	$54^{{}^{\circ}}\times 38^{{}^{\circ}}$	Image
The long-focus camera	Canon 5D Mark II	180	$11^{{}^{\circ}}\times 7.6^{{}^{\circ}}$	Image

**Table 2:** The operating Parameters of LiDAR.

Parameter	Value
Scanning speed	40 line/s
Pulse repetition rate	300 kHz
Starting scanning angle	$40^{{}^{\circ}}$
Terminational scanning angle	$105^{{}^{\circ}}$

**Table 3:** The parameters adopted in the planning flight path and task points.

Parameter	Value
Safe distance to power line in horizontal direction	30 m
Safe distance to power line in vertical direction	40 m
Safe distance of unmanned helicopter	50 m
The distance of S	50 m
The overlay of image	30%
The minimum distance for task	5 m
The minimum time for task	2 s

**Table 4:** The success rate of tasks for two camera.

Sensor	Planned Task Number	Image Number	Success Rate
The short-focus camera	163	163	100%
The long-focus camera	78	73	93.5%
Total	241	236	98%

**Table 5:** The mean and standard deviation of offset in two direction.

Direction	Mean (m)	Standard Deviation (m)
Horizontal	−0.05	0.71
Vertical	0.04	0.69
